# Supplementary material for: Asymmetric pendrin homodimer reveals its molecular mechanism as anion exchanger
Source: Nat Commun. 2023 May 25;14:3012. doi: 10.1038/s41467-023-38303-0 (PMC10213058; doi:10.1038/s41467-023-38303-0)
Supplement: Supplementary file 1 — Supplementary Information [file 41467_2023_38303_MOESM1_ESM.pdf]

Supplementary Information for

Asymmetric Pendrin Homodimer Reveals its Molecular Mechanism as Anion Exchanger

Qianying Liu[1#], Xiang Zhang[1#], Hui Huang[1#], Yuxin Chen[2,5,6,7#], Fang Wang[2,5,6,7], Aihua Hao[1], Wuqiang Zhan[1], Qiyu Mao[1], Yuxia Hu[1], Lin Han[1], Yifang Sun[1], Meng Zhang[1], Zhimin Liu[1], Genglin Li[2], Weijia Zhang[1], Yilai Shu[2,5,6,7*], Lei Sun[1,3,4*], Zhenguo Chen[1,3,4*]

Correspondence to: zhenguochen@fudan.edu.cn (Z.C.); llsun@fudan.edu.cn (L.S.); and yilai_shu@fudan.edu.cn (Y.S.)

This PDF file includes:

Supplementary Fig. 1-12

Supplementary Table 1-3

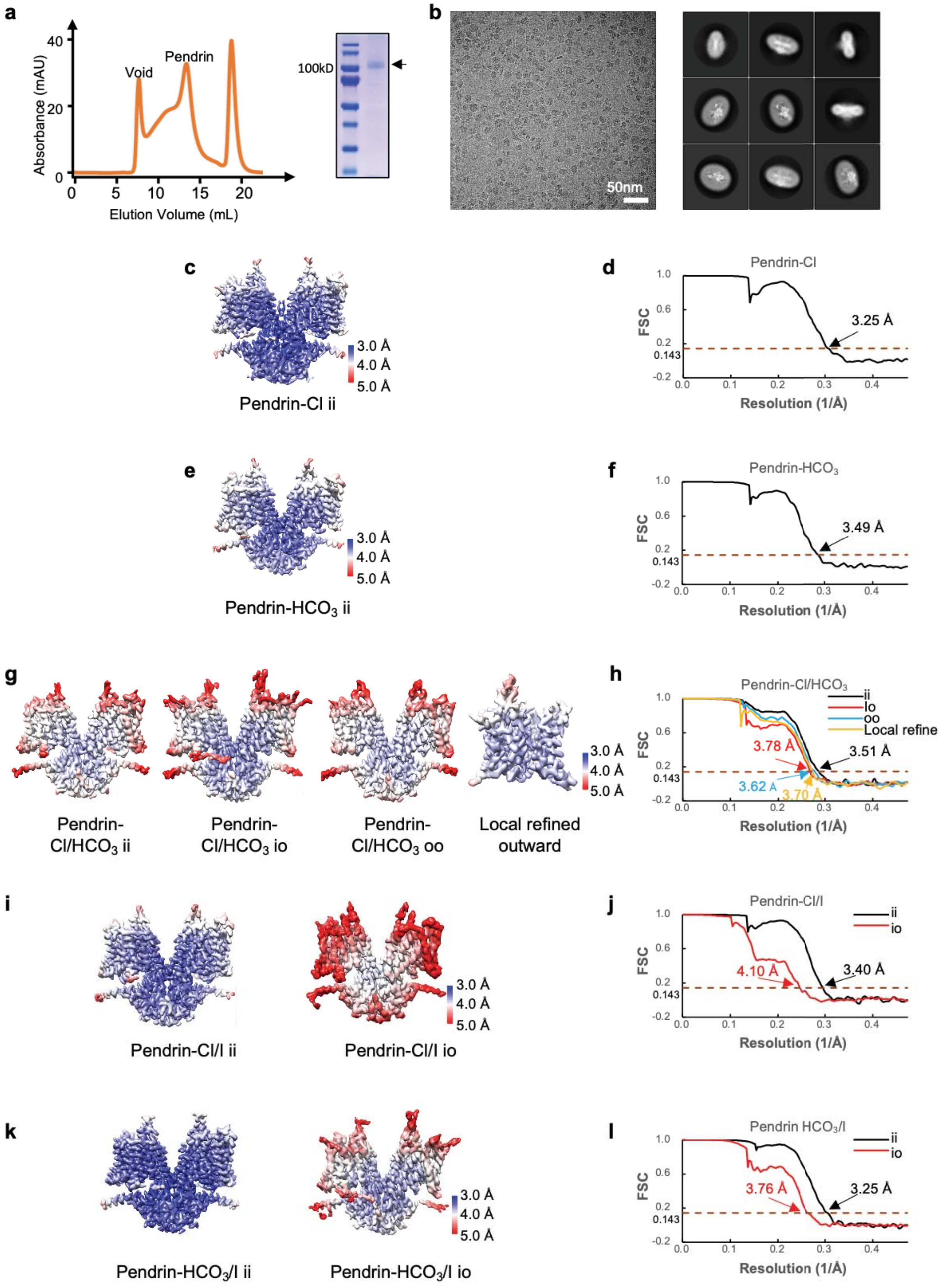

a
Absorbance (mAU)
Elution Volume (mL)
Void
Pendrin
100kD
b
50nm
c
3.0 Å
4.0 Å
5.0 Å
Pendrin-Cl ii
d
Pendrin-Cl
FSC
3.25 Å
0.143
Resolution (1/Å)
e
Pendrin-HCO3 ii
f
Pendrin-HCO3
3.49 Å
g
Pendrin-Cl/HCO3 ii
Pendrin-Cl/HCO3 io
Pendrin-Cl/HCO3 oo
Local refined outward
h
Pendrin-Cl/HCO3
ii
Io
oo
Local refine
3.78 Å
3.51 Å
3.62 Å
3.70 Å
i
Pendrin-Cl/I ii
Pendrin-Cl/I io
j
Pendrin-Cl/I
4.10 Å
3.40 Å
k
Pendrin-HCO3/I ii
Pendrin-HCO3/I io
l
Pendrin HCO3/I
3.76 Å
3.25 Å

Supplementary Fig. 1 Purification and cryo-EM data processing results of pendrin.
a, Size exclusion chromatography of pendrin-Cl and SDS-PAGE gel of the peak fraction. The features and patterns were similar among all five data sets' purification. **b,** The representative of Cryo-EM micrographs and the 2D averages from pendrin-Cl dataset. **c, e, g, i, k,** Cryo-EM maps colored according to local resolution estimations. **d, f, h, j, l,** Corresponding FSC curves of the masked cryo-EM maps. ii: inward-inward, io: inward-outward, oo: outward-outward for protomer A and protomer B, respectively.

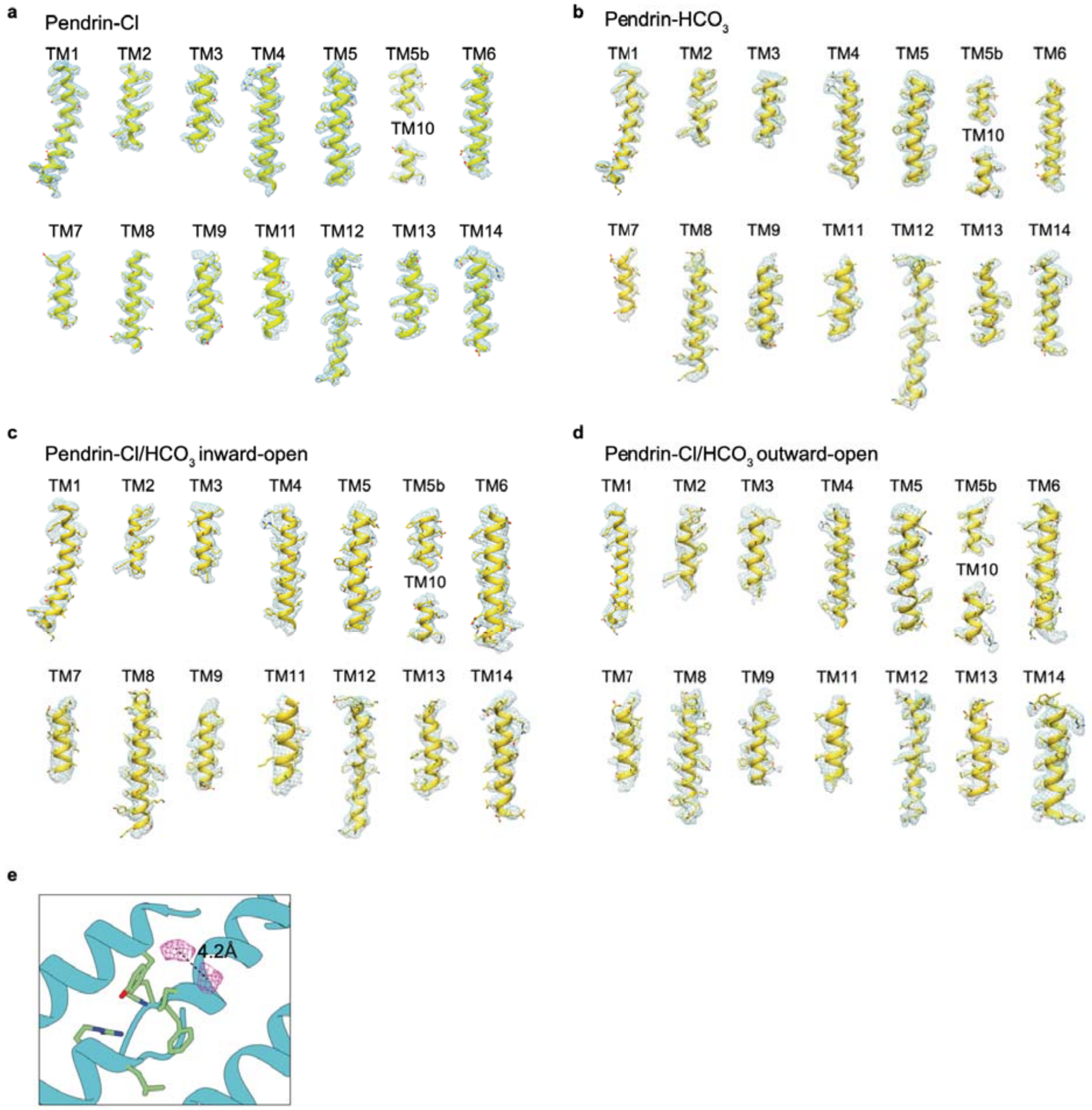

Supplementary Fig. 2 Local cryo-EM densities.

a, Representative local cryo-EM densities of pendrin-Cl_{ii}. **b,** Representative local cryo-EM densities of pendrin-$HCO_{3\,ii}$. **c** and **d,** Representative local cryo-EM densities of pendrin-$Cl/HCO_{3\,io}$. **e,** Two anion densities in the outward-open cavity in pendrin-Cl/HCO_3. The distance between two densities is labeled.

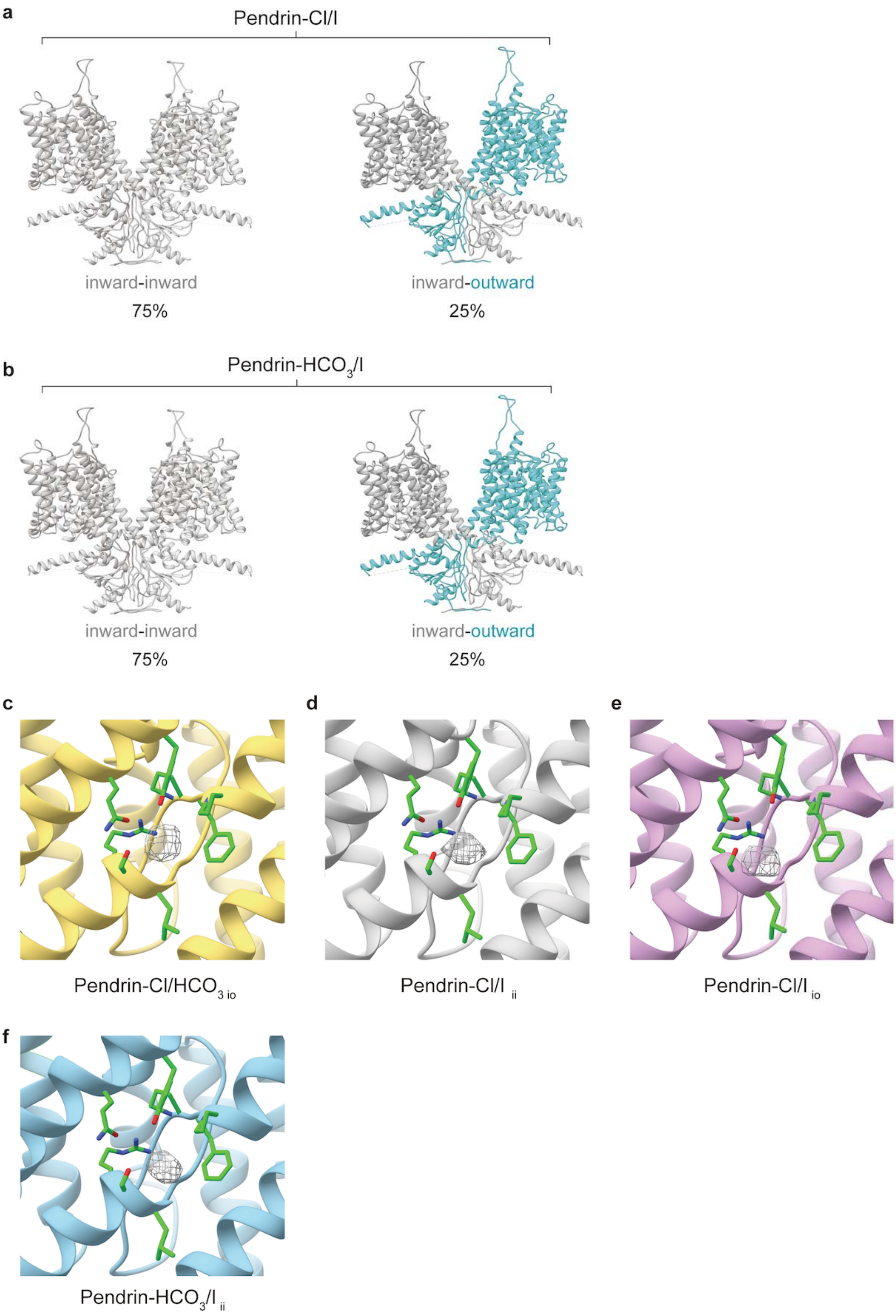
a
Pendrin-Cl/I
inward-inward
75%
inward-outward
25%
b
Pendrin-HCO3/I
inward-inward
75%
inward-outward
25%
c
Pendrin-Cl/HCO3 io
d
Pendrin-Cl/I ii
e
Pendrin-Cl/I io
f
Pendrin-HCO3/I ii

Supplementary Fig. 3 Conformations of pendrin-Cl/I and pendrin-HCO_3/I and anion densities we observed in the inward-open pocket of these structures.

a and b, Two conformations of pendrin-Cl/I and pendrin-HCO_3/I. The proportion of particles is indicated. **c-f,** Besides the pendrin-Cl and pendrin-HCO_3 in the main figure, the anion densities we absorbed in the inward-open pocket of the rest structures, c shows pendrin-Cl/$HCO_{3\ io}$, d shows pendrin-Cl/I_{ii}, e shows pendrin-Cl/I_{io} and f shows pendrin-HCO_3/I_{ii}. ii: inward-inward, io: inward-outward, oo: outward-outward for protomer A and protomer B, respectively. Density is shown in the light grey mesh.

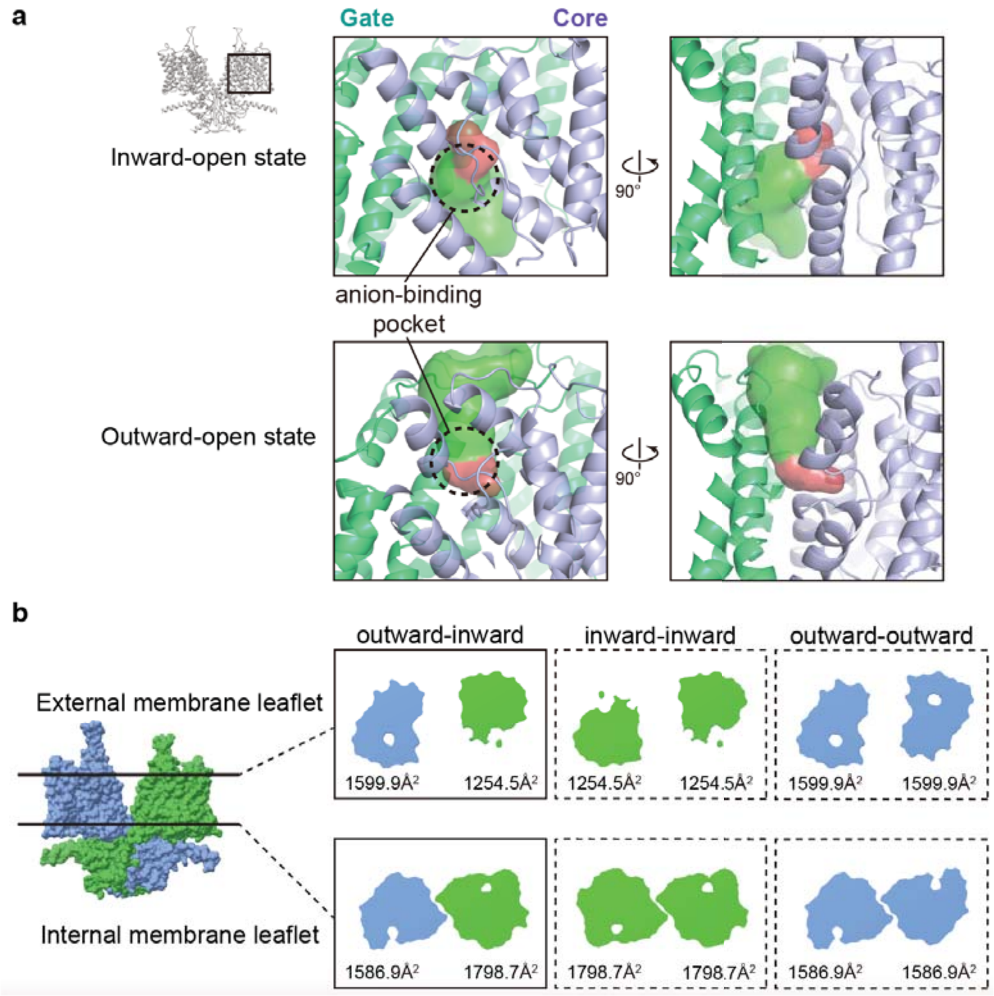

Supplementary Fig. 4 Anion pathway cavity and cross-section area of the inward-open state and the outward-open state TMDs.

Anion pathway analyzed by HOLE. Because pendrin functions in the manner of alternative accessing, we analyzed the inward-open TMD and outward-open TMD, respectively. **a,** The cavities from the anion-binding pocket to the intracellular or extracellular space (red cavity indicates the pathway diameter smaller than 3 Å, and green cavity indicates the pathway diameter larger than 3 Å). **b,** At the outer leaflet and the inner leaflet, cross-section areas of pendrin in three conformations are shown.

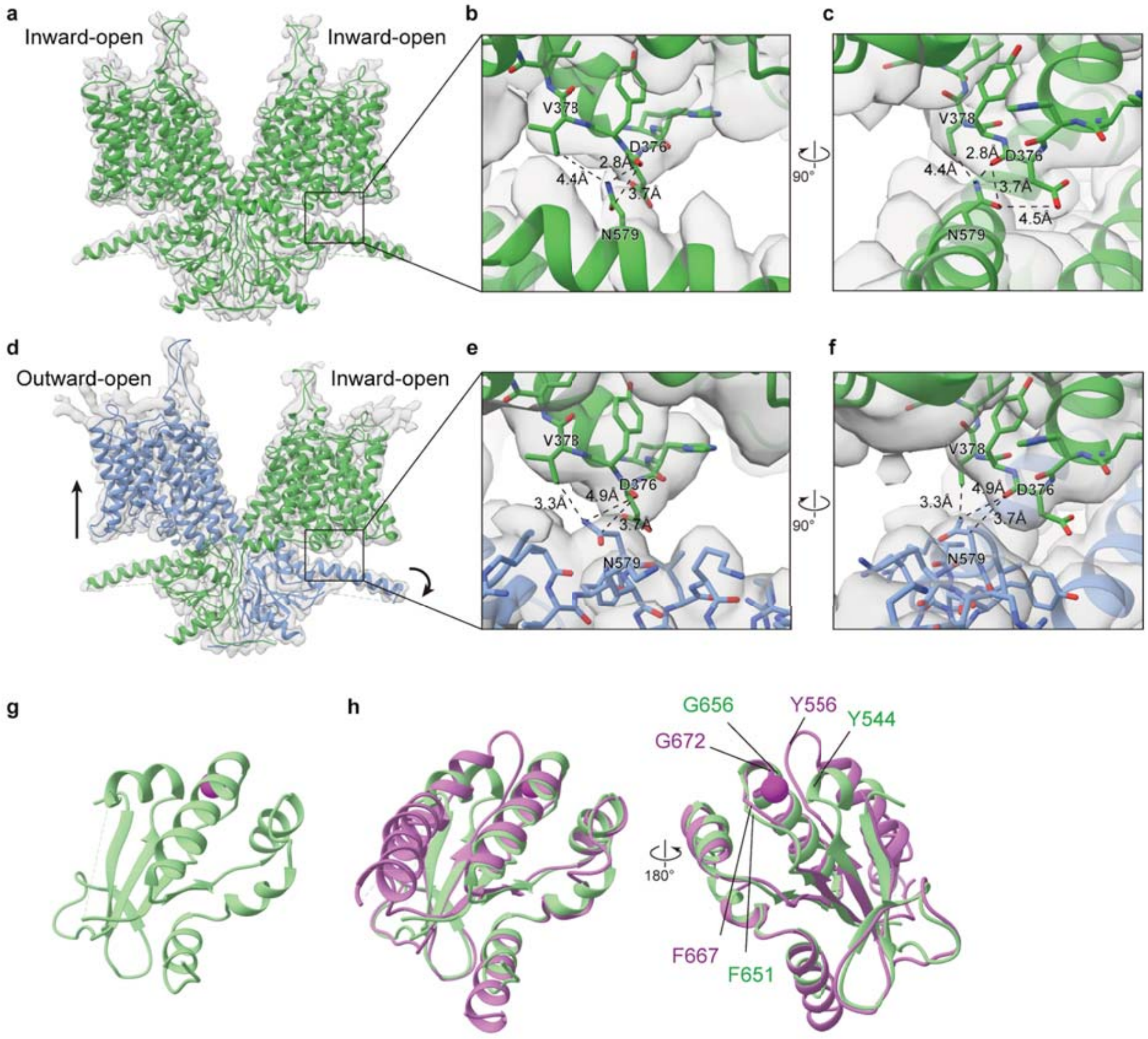

Supplementary Fig. 5 Details of the interaction between STAS domain helix Cα1b and TMD and “pre-binding site”.

a-c, In inward-open state C2 structures, the helix Cα1b is the most close to the TMD (N579 of helix Cα1b interacts with D376 of TM8 in core region). **d-f,** In the outward-open protomer of the asymmetric structures, the helix Cα1b rotates about 6° away and the core moves up to the extracellular space (marked by black arrows). Therefore, below the inward-open TMD, N579 gets a little away from D376 by Cα1b rotation. And below the outward-open TMD, interaction is broken by core’s movement. **g,** Crystal structure of rat prestin STAS domain in complex with chloride (PDBID: 5EUU). A Cl^- in the pre-binding site is shown in magenta. **h,** Comparison of rat prestin STAS domain (light green) with pendrin STAS domain (orchid). F667, G672 and Y556 of pendrin are labeled.

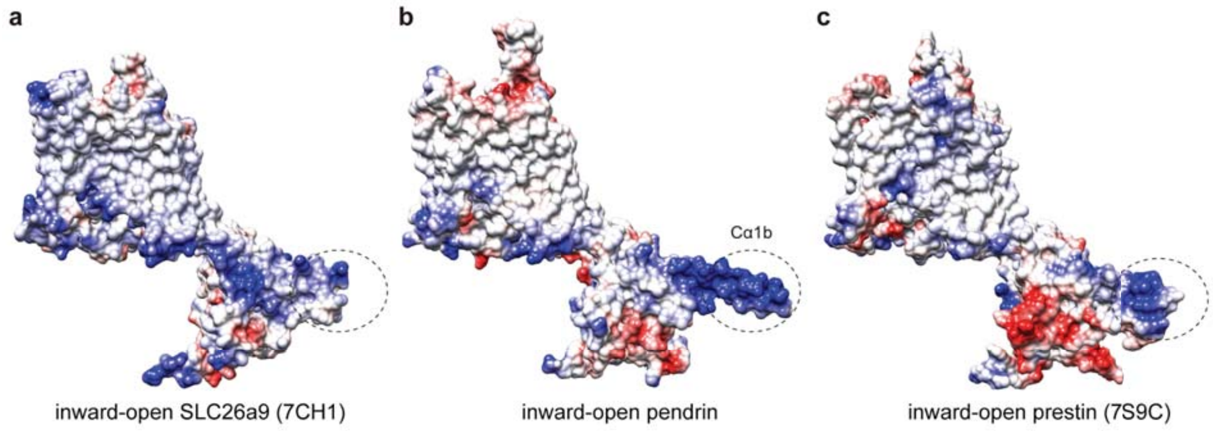

Supplementary Fig. 6 a-c, Electrostatic potential surface of pendrin, prestin, and SLC26a9 protomers, showing the positive surface charge of Cα1b.

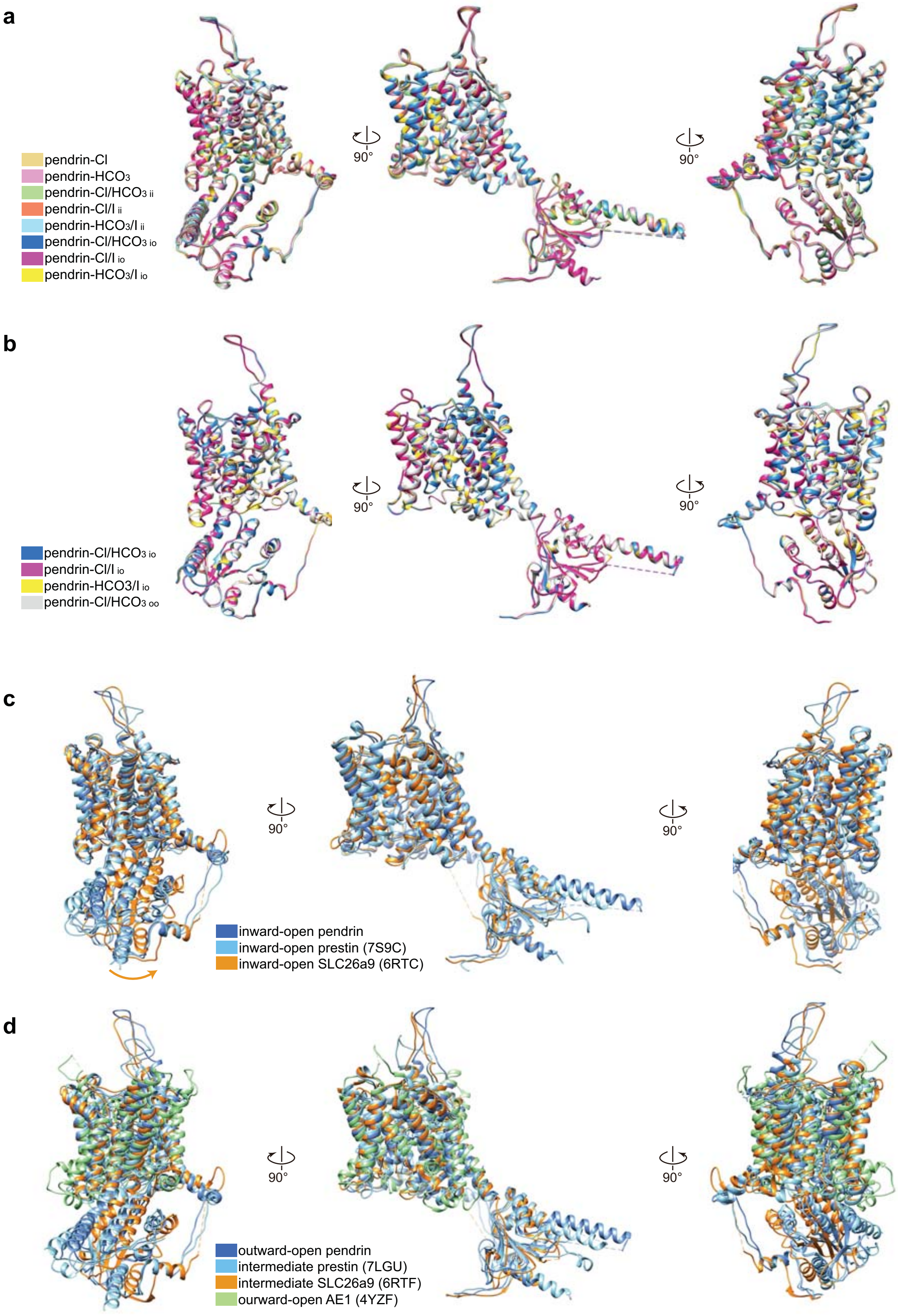

a
pendrin-Cl
pendrin-HCO3
pendrin-Cl/HCO3 ii
pendrin-Cl/I ii
pendrin-HCO3/I ii
pendrin-Cl/HCO3 io
pendrin-Cl/I io
pendrin-HCO3/I io
90°
90°
b
pendrin-Cl/HCO3 io
pendrin-Cl/I io
pendrin-HCO3/I io
pendrin-Cl/HCO3 oo
90°
90°
c
inward-open pendrin
inward-open prestin (7S9C)
inward-open SLC26a9 (6RTC)
90°
90°
d
outward-open pendrin
intermediate prestin (7LGU)
intermediate SLC26a9 (6RTF)
ourward-open AE1 (4YZF)
90°
90°

Supplementary Fig. 7 Structural comparison of pendrin, prestin, SLC26A9, and AE1.

a, Superimposing the inward-open protomers among 8 pendrin structures (pendrin-Cl, pendrin-HCO_3, pendrin pendrin-Cl/HCO_3 $_{ii}$, pendrin-Cl/I $_{ii}$, pendrin-HCO_3/I $_{ii}$, pendrin-Cl/HCO_3 $_{io}$ chain A, pendrin-Cl/I $_{io}$ chain A, pendrin-HCO_3/I $_{io}$ chain A). RMSDs are listed in Supplementary Table 1. **b,** Superimposing the outward-open protomers among 4 pendrin structures (pendrin-Cl/HCO_3 $_{io}$ chain B, pendrin-Cl/I $_{io}$ chain B, pendrin-HCO_3/I $_{io}$ chain B, pendrin-Cl/HCO_3 $_{oo}$). RMSDs are listed in Supplementary Table 1. **c,** Superimposing the inward-open protomers of pendrin-Cl, prestin (PDBID: 7S9C), SLC26A9 (PDBID: 6RTC). RMSDs are listed in Supplementary Table 2. **d,** Superimposing the outward-open protomers of pendrin-Cl/HCO_3 $_{oo}$, prestin (PDBID: 7LGU), SLC26A9 (PDBID: 6RTF), TMD of AE1 (PDBID: 4YZF). RMSDs are listed in Supplementary Table 2.

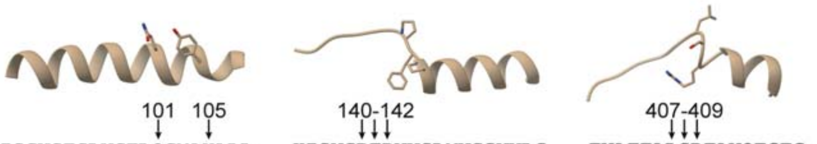


```
Q9R155|S26A4_MOUSE    ISGVSTGLVGTLQGMAYALL   HISVGPFPVVSLMVGSVVLS   FVATTALSRTAVQESTG
O43511|S26A4_HUMAN    ISGVSTGLVATLQGMAYALL   HISVGPFPVVSLMVGSVVLS   FVATTALSRTAVQESTG
Q8BU91|S26A9_MOUSE    LGGLSGGCIQVPQGMAFALL   QMVPGTFAVISILVGNICLQ   HVICCALSVTLAVDGAG
Q7LBE3|S26A9_HUMAN    LGGLSGGSIQVPQGMAFALL   QMVPGTFAVISILVGNICLQ   HVICCALSVTLAVDGAG
Q99NH7|S26A5_MOUSE    VSGISTGVLQLPQGLAFAML   HISIGPFAVISLMIGGVAVR   FSISCSLSRSLVQEGTG
P58743|S26A5_HUMAN    VSGISTGVLQLPQGLAFAML   HISIGPFAVISLMIGGVAVR   FSISCSLSRSLVQEGTG
Q9EPH0|S26A5_RAT      VSGISTGVLQLPQGLAFAML   HISIGPFAVISLMIGGVAVR   FSISCSLSRSLVQEGTG
D7PC76|D7PC76_TURTR   VSGISTGVLQLPQGLAFAML   HISIGPFAVISLMIGGVAVR   FAISCSLSRSLVQEGTG
                      :.*:* * :   **:*:*:*   ::  * * *:*:::*.: :    .    :** : . :.:*
```

Supplementary Fig. 8 SLC26A homologs sequence alignment around the anion binding pocket.

These sequences were obtained from UniProt. The entry identifier is listed along with the protein name on the left.

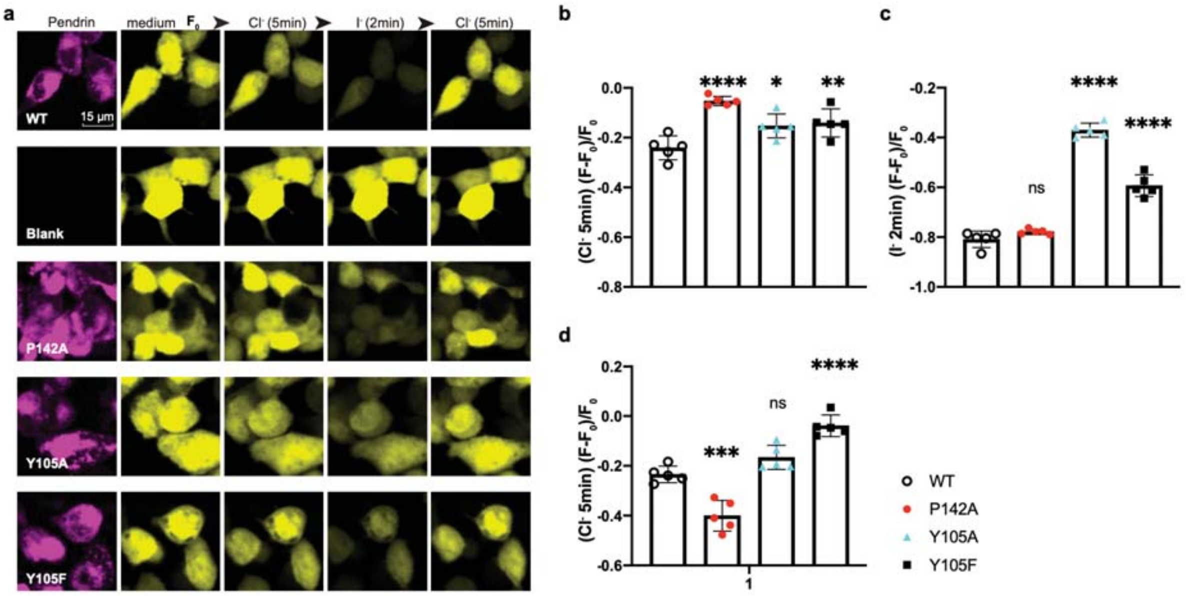

e

	Dunnett's multiple comparisons test	Mean Diff.	95.00% CI of diff.	Adjusted P Value
**Cl- 5min**	WT vs. P142A	-0.188	-0.2621 to -0.1139	<0.0001
	WT vs. Y105A	-0.08795	-0.1621 to -0.01384	0.019
	WT vs. Y105F	-0.1001	-0.1742 to -0.02601	0.0079
**I- 2 min**	WT vs. P142A	-0.03049	-0.08163 to 0.02064	0.314
	WT vs. Y105A	-0.4386	-0.4898 to -0.3875	<0.0001
	WT vs. Y105F	-0.2158	-0.2670 to -0.1647	<0.0001
**Cl- 5 min**	WT vs. P142A	0.166	0.08747 to 0.2446	0.0002
	WT vs. Y105A	-0.06853	-0.1471 to 0.01004	0.0937
	WT vs. Y105F	-0.1954	-0.2739 to -0.1168	<0.0001

Supplementary Fig. 9 Fluorescence change of HEK293T cells in Cl^-/I^- exchange assay.

a, Enlargement of raw fluorescence micrographs. **b-d,** At three time points, One-way ANOVA with Dunnett's multiple comparison test was performed for comparison between multiple groups. n= 5 cells examined over 3 independent experiments. Data are presented as mean values, error bars indicate SD. Multiple comparison results were the comparison versus the cells transfected with WT pendrin, ****P < 0.0001, ***P < 0.001, **P < 0.01, and *P < 0.05. **e.** Details of Dunnett's multiple comparisons test.

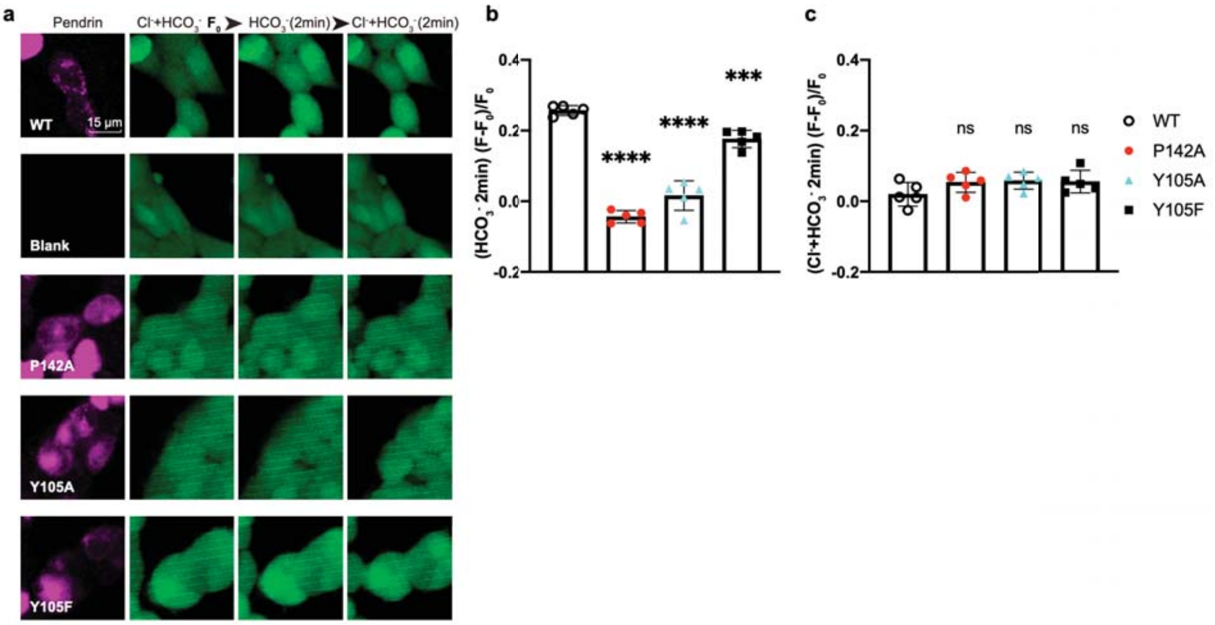

d

	Dunnett's multiple comparisons test	Mean Diff.	95.00% CI of diff.	Adjusted P Value
HCO_3^- 2min	WT vs. P142A	0.3006	0.2568 to 0.3444	<0.0001
	WT vs. Y105A	0.2407	0.1968 to 0.2845	<0.0001
	WT vs. Y105F	0.08033	0.03650 to 0.1242	0.0006
Cl^-+HCO_3^- 2min	WT vs. P142A	-0.03364	-0.08223 to 0.01494	0.2121
	WT vs. Y105A	-0.03836	-0.08695 to 0.01022	0.1381
	WT vs. Y105F	-0.03473	-0.08331 to 0.01385	0.1927

Supplementary Fig. 10 Fluorescence change of HEK293T cells in Cl^-/HCO_3^- exchange assay.
a, Enlargement of raw fluorescence micrographs. **b-c,** At two time points, One-way ANOVA with Dunnett's multiple comparison test was performed for comparison between multiple groups. n= 5 cells examined over 3 independent experiments. Data are presented as mean values, error bars indicate SD. Multiple comparison results were the comparison versus the cells transfected with WT pendrin, ****P < 0.0001, ***P < 0.001, **P < 0.01, and *P < 0.05. **d.** Details of Dunnett's multiple comparisons test.

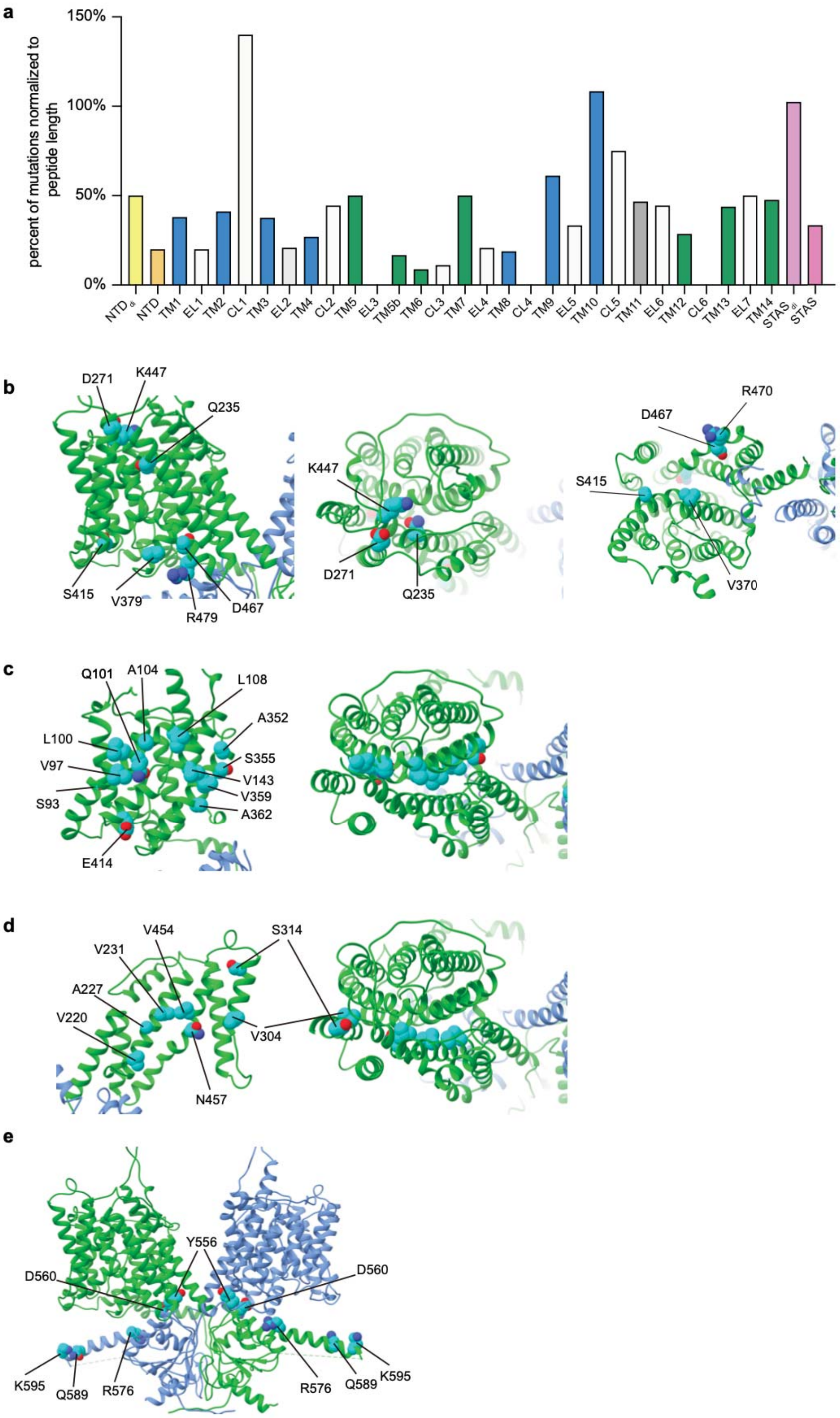
a
150%
100%
50%
0%
percent of mutations normalized to peptide length
NTD d
NTD
TM1
EL1
TM2
CL1
TM3
EL2
TM4
CL2
TM5
EL3
TM5b
TM6
CL3
TM7
EL4
TM8
CL4
TM9
EL5
TM10
CL5
TM11
EL6
TM12
CL6
TM13
EL7
TM14
STAS d
STAS
b
D271
K447
Q235
S415
V379
R479
D467
K447
D271
Q235
R470
D467
S415
V370
c
Q101
A104
L108
A352
L100
S355
V97
V143
V359
S93
A362
E414
d
V454
S314
V231
A227
V220
V304
N457
e
Y556
D560
D560
K595
Q589
R576
R576
Q589
K595

Supplementary Fig. 11 Summary of 261 pathogenetic mutations of pendrin.
a, Based on the deafness variation database (https://deafnessvariationdatabase.org; accessed on 20 Sep 2022), 261 of 761 missense disease-associated mutations are pathogenetic in clinic. NTD_{di} indicates the residue in the dimerization interface of NTD, EL indicates the extracellular loop, CL indicates the cytosolic loop, and $STAS_{di}$ indicates the residue in the dimerization interface of STAS. **b,** Mutations along the anion exchange pathway that would change local charge. **c,** In the core region, mutation locations happened to change the interface profile. **d,** In the gate region, mutations are located on the core-gate interface regions. **e,** On the positively charged platform, the mutation locations happened to change charge.

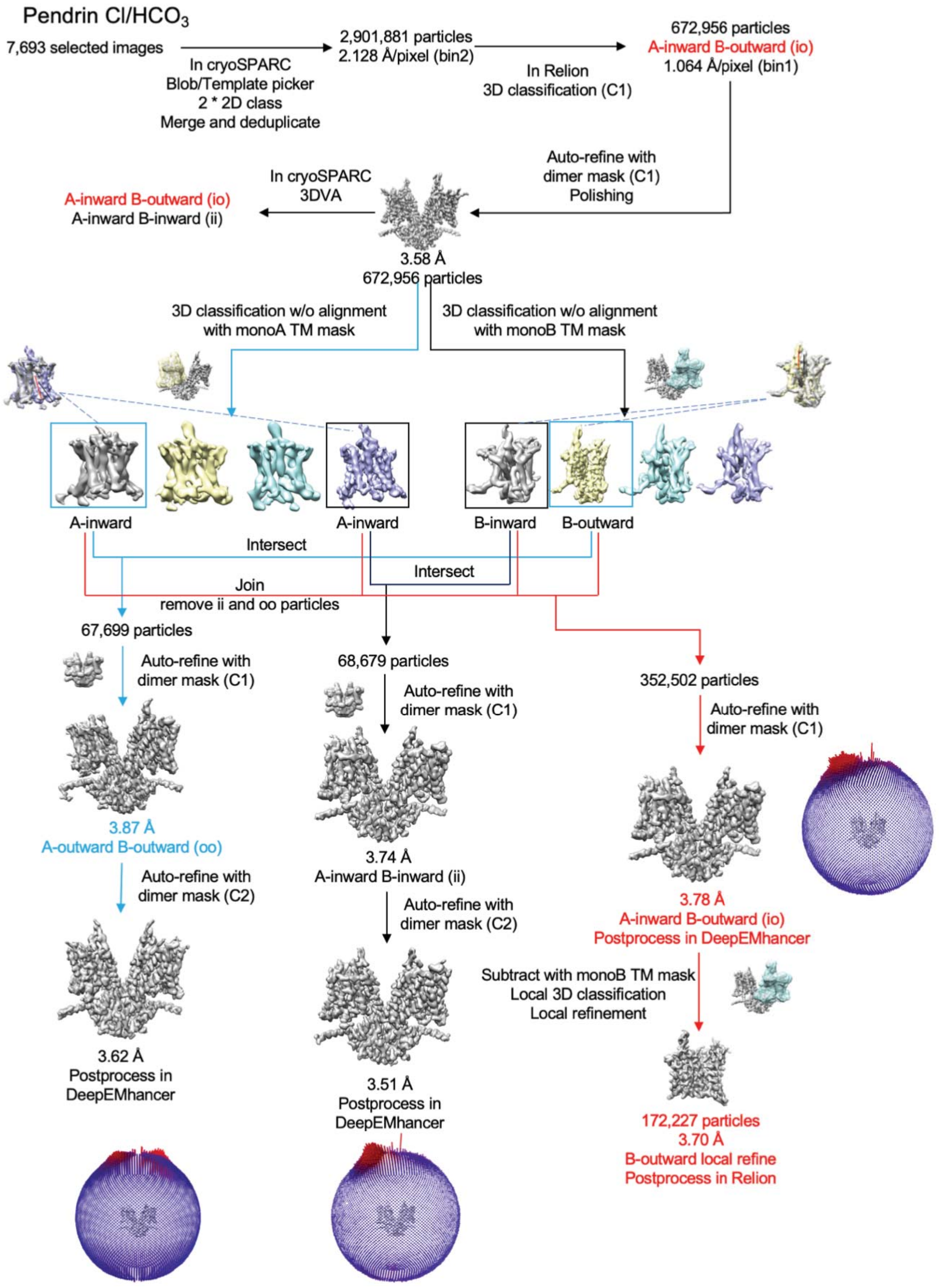

Supplementary Fig. 12 Flowchart of pendrin-Cl/HCO_3 data processing.

Supplementary Table 1 Comparison of pendrin structures at different conditions (RMSD).

pendrin-Cl												
Pendrin-HCO_3	0.3 (Å)											
pendrin-Cl/HCO_3 ii	0.4	0.4										
pendrin-Cl/I ii	0.4	0.4	0.4									
pendrin-HCO_3/I ii	0.4	0.4	0.4	0.3								
pendrin-Cl/HCO_3 io A	0.5	0.5	0.4	0.5	0.5							
pendrin-Cl/HCO_3 io B	4.4	4.5	4.4	4.4	4.4	4.4						
pendrin-Cl/I io A	0.5	0.5	0.4	0.4	0.5	0.2	4.3					
pendrin-Cl/I io B	4.4	4.5	4.3	4.3	4.3	4.3	0.2	4.3				
pendrin-HCO_3/I io A	0.5	0.5	0.5	0.5	0.5	0.2	4.4	0.3	4.4			
pendrin-HCO_3/I io B	4.3	4.4	4.3	4.3	4.3	4.3	0.2	4.3	0.2	4.4		
pendrin-Cl/HCO_3 oo	4.5	4.6	4.4	4.4	4.5	4.4	0.3	4.4	0.4	4.5	0.4	
	pendrin-Cl	pendrin-HCO_3	pendrin-Cl/HCO_3 ii	pendrin-Cl/I ii	pendrin-HCO_3/I ii	pendrin-Cl/HCO_3 io A	pendrin-Cl/HCO_3 io B	pendrin-Cl/I io A	pendrin-Cl/I io B	pendrin-HCO_3/I io A	pendrin-HCO_3/I io B	pendrin-Cl/HCO_3 oo

Supplementary Table 2 Comparison of pendrin structures with representative structures of prestin and SLC26A9 (RMSD).

Pendrin-Cl										
SLC26A9 (6RTC)	4.6 (Å)									
SLC26A9 (7CH1)	4.1	3.4								
Prestin (7S9C)	3	5.3	5.3							
Prestin (7LH2)	3.8	6.3	6.3	2.3						
Prestin (7S9D)	3.1	6.1	6	2.1	1.9					
Prestin (7S8X)	3.6	6.9	7.2	2.7	1.6	1.9				
Prestin (7LGU)	4.5	7.5	8	3.1	1.2	2.4	1.4			
SLC26A9 (6RTF)	4.3	3	4.8	4.3	4.4	4.8	5.1	5.8		
Pendrin-Cl/HCO_3 oo	4.5	7	6.8	4.4	4.2	3.8	3.3	3.9	5.1	
	Pendrin-Cl	SLC26A9(6RTC)	SLC26A9(7CH1)	Prestin (7S9C)	Prestin (7LH2)	Prestin (7S9D)	Prestin (7S8X)	Prestin (7LGU)	SLC26A9(6RTF))	Pendrin-Cl/HCO_3 oo

Supplementary Table 3 Cryo-EM data collection and refinement statistics.

	pendrin-Cl	pendrin-HCO_3	pendrin-Cl/I_{ii}	pendrin-Cl/I_{io}	pendrin-$Cl/HCO_{3\,ii}$	pendrin-$Cl/HCO_{3\,io}$	Local refinement of pendrin-$Cl/HCO_{3\,io}$	pendrin-$Cl/HCO_{3\,oo}$	pendrin-HCO_3/I_{ii}	pendrin-HCO_3/I_{io}
**PDB ID**	7WK1	7WK7	7WL8	7WLB	7WL7	7WL9		7WLE	7WL2	7WLA
**EMDB**	32555	32561	32577	32580	32576	32578	33232	32583	32574	32579
**Data collection and processing**										
Data sets	pendrin-Cl	pendrin-HCO_3	pendrin-Cl/I		pendrin-Cl/HCO_3				pendrin- HCO_3/I	
Voltage (kV)	300	300	300		300				300	
Detector	K2	K2	K3		K3				K2	
Pixel size (Å)	1.046	1.046	1.064		1.064				1.046	
Electron dose ($e^-/Å^2$)	53	53	58		58				53	
Defocus range (μm)	−1.2 to −2.2	−1.2 to −2.2	−1.2 to −2.2		−1.2 to −2.2				−1.2 to −2.2	
Final particles	120,887	134,404	79,043	45,859	68,679	353,288	172,227	67,699	221,446	365,679
Final resolution (Å)	3.25	3.49	3.40	4.10	3.51	3.78	3.70	3.62	3.25	3.76
**Model refinement and validation statistics**										
Ramachandran statistics										
Favored (%)	94.70	94.93	95.31	95.45	95.31	95.30		93.92	95.01	94.92
Allowed (%)	5.22	4.99	4.61	4.55	4.61	4.62		6.08	4.92	5.08
Outliers (%)	0.08	0.08	0.08	0.00	0.08	0.08		0.00	0.08	0.00
Rotamer outliers (%)	3.12	2.94	1.87	3.92	3.39	4.27		5.25	2.14	4.01
R.m.s.deviations										
Bond lengths (Å)	0.002	0.001	0.002	0.002	0.001	0.002		0.002	0.002	0.002
Bond angles (°)	0.406	0.406	0.402	0.400	0.409	0.403		0.426	0.412	0.405
