# Supplementary material for: Asymmetric pendrin homodimer reveals its molecular mechanism as anion exchanger
Source: Nat Commun. 2023 May 25;14:3012. doi: 10.1038/s41467-023-38303-0 (PMC10213058; doi:10.1038/s41467-023-38303-0)
Supplement: Supplementary file 2 — Description of Additional Supplementary Files [file 41467_2023_38303_MOESM2_ESM.pdf]

Description of Additional Supplementary Files

File Name: Supplementary Data 1
Description: Pathogenetic missense variants of pendrin from Deafness Variation Database.

File Name: Supplementary Movie 1
Description: Conformational change of pendrin homodimer. The movement of the inward-open state to the outward-open state is simulated.
