# Supplementary material for: Asymmetric pendrin homodimer reveals its molecular mechanism as anion exchanger
Source: Nat Commun. 2023 May 25;14:3012. doi: 10.1038/s41467-023-38303-0 (PMC10213058; doi:10.1038/s41467-023-38303-0)
Supplement: Supplementary file 5 — Reporting Summary [file 41467_2023_38303_MOESM5_ESM.pdf]

nature portfolio

Corresponding author(s): Zhenguo Chen

Last updated by author(s): Jan 21, 2023

Reporting Summary

Nature Portfolio wishes to improve the reproducibility of the work that we publish. This form provides structure for consistency and transparency in reporting. For further information on Nature Portfolio policies, see our Editorial Policies and the Editorial Policy Checklist.

Statistics

For all statistical analyses, confirm that the following items are present in the figure legend, table legend, main text, or Methods section.

n/a	Confirmed	
☐	☒	The exact sample size (*n*) for each experimental group/condition, given as a discrete number and unit of measurement
☐	☒	A statement on whether measurements were taken from distinct samples or whether the same sample was measured repeatedly
☐	☒	The statistical test(s) used AND whether they are one- or two-sided<br>*Only common tests should be described solely by name; describe more complex techniques in the Methods section.*
☐	☒	A description of all covariates tested
☐	☒	A description of any assumptions or corrections, such as tests of normality and adjustment for multiple comparisons
☐	☒	A full description of the statistical parameters including central tendency (e.g. means) or other basic estimates (e.g. regression coefficient) AND variation (e.g. standard deviation) or associated estimates of uncertainty (e.g. confidence intervals)
☐	☒	For null hypothesis testing, the test statistic (e.g. *F*, *t*, *r*) with confidence intervals, effect sizes, degrees of freedom and *P* value noted<br>*Give P values as exact values whenever suitable.*
☐	☒	For Bayesian analysis, information on the choice of priors and Markov chain Monte Carlo settings
☐	☒	For hierarchical and complex designs, identification of the appropriate level for tests and full reporting of outcomes
☐	☒	Estimates of effect sizes (e.g. Cohen's *d*, Pearson's *r*), indicating how they were calculated

Our web collection on statistics for biologists contains articles on many of the points above.

Software and code

Policy information about availability of computer code

Data collection

All softwares and algorithms used in this study are open source.
Cryo-EM data collection: Some pendrin cryo-EM datasets (including pendrin-Cl, pendrin-HCO3 and pendrin-HCO3/I) were collected on Titan Krios (Thermo Fisher Scientific®) operated at 300 kV, equipped with K2 Summit direct electron detection device (Gatan®) and BioQuantum energy filter (Gatan®) set to a slit width of 20 eV. Automated data acquisition was carried out with SerialEM software through the beam-image shift method. Others (pendrin-Cl/HCO3, pendrin-Cl/I) were collected on the same microscope with the camera upgraded to K3 Summit (Gatan®) using a slit width of 20 eV.
Fluorescence anion exchange assay: Fluorescence was recorded at the excitation wavelengths of 488 nm and the emission wavelengths of 530±10 nm by confocal microscope (Leica TCS SP8).

Data analysis

All softwares and algorithms used in this study are open source.
Cryo-EM image processing: Movie stacks were binned 2×2, dose weighted, and motion corrected using MotionCor2 within RELION3.0. Parameters of contrast transfer function (CTF) were estimated by using Gctf. RELION3.0 and cryoSPARC were used for 3D reconstruction. Sharpened maps were generated by DeepEMhancer. All the visualization and evaluation of 3D density maps were performed with UCSF Chimera.
Model building and structure refinement: Model building of inward-open state with C2 symmetry was refined from predicted model in Alpha Fold with Coot. Structure refinement was performed with PHENIX.
Structure analysis: Anion transport pathway was analysed by HOLE v2.2.005.
Fluorescence anion exchange assay: The fluorescence intensity was measured by ImageJ 1.53a. The fluorescence intensity was calculated by GraphPad Prism 8. We defined the fluorescence intensity in medium after 10 min as F0 and drew the (F- F0)/ F0 curves (n=5 cells examined over 3 independent experiments, curve data points indicate mean, error bars indicate SD). One-way ANOVA with Dunnett's multiple

comparison test was performed for comparison between multiple groups. Multiple comparison results were the comparison versus the cells transfected with WT pendrin.

For manuscripts utilizing custom algorithms or software that are central to the research but not yet described in published literature, software must be made available to editors and reviewers. We strongly encourage code deposition in a community repository (e.g. GitHub). See the Nature Portfolio guidelines for submitting code & software for further information.

Data

Policy information about availability of data

All manuscripts must include a data availability statement. This statement should provide the following information, where applicable:

- Accession codes, unique identifiers, or web links for publicly available datasets
- A description of any restrictions on data availability
- For clinical datasets or third party data, please ensure that the statement adheres to our policy

The cryo-EM density maps and coordinates have been deposited in the Electron Microscopy Data Bank (EMDB) and Protein Data Bank (PDB), respectively, with accession codes 32555 and 7WK1 for pendrin-Cl, 32561 and 7WK7 for pendrin-HCO3, 32577 and 7WL8 for pendrin-Cl/I ii, 32580 and 7WLB for pendrin-Cl/I io, 32576 and 7WL7 for pendrin-Cl/HCO3 ii, 32578 and 7WL9 for pendrin-Cl/HCO3 io, 32583 and 7WLE for pendrin-Cl/HCO3 oo, 32574 and 7WL2 for pendrin-HCO3/I ii, 32579 and 7WLA for pendrin-HCO3/I io. The local refinement map of pendrin-Cl/HCO3 io has EMDB code 33232.
And for structures mentioned in this study, with PDB accession codes for 7S9C for inward-open prestin, 6RTC for inward-open SLC26A9, 7LGU for intermediate prestin, 6RTF for intermediate SLC26A9, 4YZF for outward-open AE1.

Human research participants

Policy information about studies involving human research participants and Sex and Gender in Research.

Reporting on sex and gender	N/A
Population characteristics	N/A
Recruitment	N/A
Ethics oversight	N/A

Note that full information on the approval of the study protocol must also be provided in the manuscript.

Field-specific reporting

Please select the one below that is the best fit for your research. If you are not sure, read the appropriate sections before making your selection.

☒ Life sciences ☐ Behavioural & social sciences ☐ Ecological, evolutionary & environmental sciences

For a reference copy of the document with all sections, see nature.com/documents/nr-reporting-summary-flat.pdf

Life sciences study design

All studies must disclose on these points even when the disclosure is negative.

Sample size	Sample sizes for cryo-EM study is were estimated based on the guidelines from instrument manufacturer.<br>For fluorescence anion exchange assays, since Fluorescence anion exchange assays were performed with n=3 independent biological replicates and attempts at replication were successful. "n=5 cells examined over 3 independent experiments" was chosen for data statistics.
Data exclusions	Bad particle images were excluded during Cryo-EM data processing because of excessive movement, low defocus, high defocus, or over-focus. No data points were excluded in fluorescence anion exchange assay.
Replication	Single particle cryo-EM is based on averaging protein particles of more or less identical orientation in a vitreous thin ice layer. Accordingly, replication is not required to ensure statistical robustness. Fluorescence anion exchange assays were performed with n=3 independent biological replicates and attempts at replication were successful.
Randomization	Samples were not allocated into experimental groups in cryo-EM study because the goal of this study was not to evaluate the impact of a particular factor, but to simply determine the structure of a protein. Allocation in fluorescence anion exchange assay was randomized.
Blinding	N/A. There was no existing protein structure to refer to and the data analysis was ab initio.

Reporting for specific materials, systems and methods

We require information from authors about some types of materials, experimental systems and methods used in many studies. Here, indicate whether each material, system or method listed is relevant to your study. If you are not sure if a list item applies to your research, read the appropriate section before selecting a response.

Materials & experimental systems

n/a	Involved in the study
☒	☐ Antibodies
☐	☒ Eukaryotic cell lines
☒	☐ Palaeontology and archaeology
☒	☐ Animals and other organisms
☒	☐ Clinical data
☒	☐ Dual use research of concern

Methods

n/a	Involved in the study
☒	☐ ChIP-seq
☒	☐ Flow cytometry
☒	☐ MRI-based neuroimaging

Eukaryotic cell lines

Policy information about cell lines and Sex and Gender in Research

Cell line source(s)	Cell lines used in this study are commercial.<br>Sf9 (RRID:CVCL_0549) is from express system®, 94-001F.<br>HEK293-EBNA (RRID:CVCL_6974) is from Procell Life Science&Technology®, CL-0311.<br>HEK293T (RRID:CVCL_0063) is from Thermo Fisher Scientific®, NC0260915.
Authentication	Cell lines used were not authenticated in the lab.
Mycoplasma contamination	Cell lines used were not tested for mycoplasma contamination.
Commonly misidentified lines<br>(See ICLAC register)	No commonly misidentified lines were used.
